# Accuracy of Radiomics in Predicting *IDH* Mutation Status in Diffuse Gliomas: A Bivariate Meta-Analysis

**DOI:** 10.1148/ryai.220257

**Published:** 2023-12-06

**Authors:** Gianfranco Di Salle, Lorenzo Tumminello, Maria Elena Laino, Sherif Shalaby, Gayane Aghakhanyan, Salvatore Claudio Fanni, Maria Febi, Jorge Eduardo Shortrede, Mario Miccoli, Lorenzo Faggioni, Mirco Cosottini, Emanuele Neri

**Affiliations:** From Academic Radiology, Department of Translational Research on New Technologies in Medicine and Surgery (G.D.S., L.T., G.A., S.C.F., M.F., J.E.S., L.F., E.N.), Department of Clinical and Experimental Medicine (M.M.), and Neuroradiology Unit, Department of Translational Research on New Technologies in Medicine and Surgery (M.C.), University of Pisa, Via Roma 67, 56126 Pisa, Italy; Artificial Intelligence Center, IRCCS Humanitas Research Hospital, Rozzano, Milan, Italy (M.E.L.); The Shrewsbury and Telford Hospital NHS Trust, Shrewsbury, England (S.S.); and Italian Society of Medical and Interventional Radiology, SIRM Foundation, Milan, Italy (E.N.).

**Keywords:** Neuro-Oncology, Radiomics, Integration, Application Domain, Glioblastoma, IDH Mutation, Radiomics Quality Scoring

## Abstract

**Purpose:**

To perform a systematic review and meta-analysis assessing the predictive accuracy of radiomics in the noninvasive determination of isocitrate dehydrogenase *(IDH*) status in grade 4 and lower-grade diffuse gliomas.

**Materials and Methods:**

A systematic search was performed in the PubMed, Scopus, Embase, Web of Science, and Cochrane Library databases for relevant articles published between January 1, 2010, and July 7, 2021. Pooled sensitivity and specificity across studies were estimated. Risk of bias was evaluated using Quality Assessment of Diagnostic Accuracy Studies-2, and methods were evaluated using the radiomics quality score (RQS). Additional subgroup analyses were performed according to tumor grade, RQS, and number of sequences used (PROSPERO ID: CRD42021268958).

**Results:**

Twenty-six studies that included 3280 patients were included for analysis. The pooled sensitivity and specificity of radiomics for the detection of *IDH* mutation were 79% (95% CI: 76, 83) and 80% (95% CI: 76, 83), respectively. Low RQS scores were found overall for the included works. Subgroup analyses showed lower false-positive rates in very low RQS studies (RQS < 6) (meta-regression, *z* = -1.9; *P* = .02) compared with adequate RQS studies. No substantial differences were found in pooled sensitivity and specificity for the pure grade 4 gliomas group compared with the all-grade gliomas group (81% and 86% vs 79% and 79%, respectively) and for studies using single versus multiple sequences (80% and 77% vs 79% and 82%, respectively).

**Conclusion:**

The pooled data showed that radiomics achieved good accuracy performance in distinguishing *IDH* mutation status in patients with grade 4 and lower-grade diffuse gliomas. The overall methodologic quality (RQS) was low and introduced potential bias.

**Keywords:** Neuro-Oncology, Radiomics, Integration, Application Domain, Glioblastoma, IDH Mutation, Radiomics Quality Scoring

*Supplemental material is available for this article.*

Published under a CC BY 4.0 license.

**Summary**

In this meta-analysis of 26 studies with 3280 patients, radiomics techniques achieved good accuracy performance in distinguishing isocitrate dehydrogenase mutation status in patients with grade 2–4 gliomas.

**Key Points**

■ According to a meta-analysis of 26 studies, pooled sensitivity and specificity achieved in distinguishing isocitrate dehydrogenase mutation status in patients with diffuse gliomas were 81% and 79%, respectively.
■ Low radiomics quality scores (RQS) were found overall (mean = 10.6 ± 3.3 [SD]), and it was observed that very low RQS scores influenced diagnostic accuracy metrics (false-positive rates meta-regression, *z* = -1.9; *P* = .02), leading to substantial overestimation.
■ Subgroup analyses showed no evidence of differences in pooled sensitivity and specificity for the grade 4 gliomas group compared with the all-grade gliomas group (*z* = 0.125 and -0.013; *P* = .90 and .99, respectively).

## Introduction

Glioblastoma (GBM) is a highly lethal brain tumor and the most common and aggressive among diffuse gliomas. It belongs to class 4 in the World Health Organization classification of brain tumors, which includes the most biologically aggressive types that have inherent heterogeneity in histopathology, microscopic anatomy, and genetic features (1,2). GBM exhibits substantial genetic heterogeneity, with various genes associated with the disease (3) and others dividing patients into different prognostic subgroups according to their methylation status (4,5).

The mutation of isocitrate dehydrogenase (*IDH*), present in approximately 12% of grade 4 glioma cases, was initially considered a prognostic factor for GBM (6). It has been found to be associated with longer overall survival and better response to chemotherapy with temozolomide compared with patients with wild-type* IDH1* or *IDH2* (7). Thus, *IDH*-mutated grade 4 gliomas were classified as a separate category of GBM in the World Health Organization 2016 Classification of Tumors of the Central Nervous System and as astrocytomas in the 2021 update. Genotyping for *IDH* is therefore essential for diagnostic workup and prognostic evaluation of patients with high-grade gliomas and may play a role in selecting patients for targeted therapies. Indeed, preliminary evidence is emerging about *IDH*-specific therapeutic interventions in patients with gliomas (8–10), increasing the need for in vivo mutational assessment. However, noninvasive techniques for *IDH* genotyping are currently lacking, limiting their application in preoperative settings.

In recent years, radiomics has emerged as a quantitative approach of artificial intelligence for the analysis of imaging data, enabling the extraction of numerous features and their correlation with clinical information, potentially revolutionizing clinical decision-making (11). Recently, mathematical analysis instruments in radiomics have been used in multiple endeavors to noninvasively determine *IDH* mutation status in diffuse gliomas, primarily relying on baseline MRI and occasionally other imaging techniques. An initial comprehensive analysis of these studies was conducted in early 2020 (12), predating the publication of the revised glioma classification. Subsequently, the volume of evidence on this topic has more than doubled, indicating the necessity for an updated overview. In this study, we conducted a state-of-the-art systematic review and meta-analysis to assess the predictive accuracy of radiomics in the noninvasive determination of *IDH* mutation status in grade 4 and lower-grade diffuse gliomas.

## Materials and Methods

### Database Search Strategy

We performed a systematic search of PubMed, Scopus, Embase, Web of Science, and Cochrane Library databases for articles relevant to the application of radiomics in the noninvasive determination of *IDH* status in grade 4 gliomas. Details on the string used in the search can be found in Appendix S1. All reviews were performed according to the Preferred Reporting Items for Systematic Reviews and Meta-Analyses, or PRISMA, 2020 guidelines (13). Since we expected heterogeneity of studies and low level of available evidence, we did not formulate PICO (population, intervention, comparison, and outcome) questions. This review was registered in the PROSPERO database (ID: CRD42021268958). All original research articles reporting on humans, written and published (including those distributed online first) from 2016 to July 2021 that respected the following criteria, were included: English language; studies including MRI, PET, or CT as imaging techniques; patients with a diagnosis of GBM; specified number of patients; and pathologic results proven with either surgery or biopsy. Exclusion criteria included review articles, conference papers and editorials or commentaries; patients younger than 18 years old; patients previously treated with surgery or radiation therapy; language other than English; no GBM diagnosis; no radiomics applications; no *IDH* mutation detection; and no statistical models for *IDH* mutational status assessment.

Importantly, the time lapse considered for inclusion of articles exactly matches the validity period of the World Health Organization 2016 classification. As the nomenclature of gliomas in publications was adapted to the World Health Organization 2016 classification, the search string was also worded accordingly.

To avoid neglecting literature data about GBM, we chose to include studies that had mixed cohorts of grade 2–4 gliomas in our meta-analysis when data about GBM were not dissociable from the rest of the cohort. A more conservative, grade 4–only subgroup meta-analysis was further performed (see below and Appendix S1).

First, two reviewers (radiologists G.D.S. with 2 years of experience and S.S. with 7 years of experience) independently screened the titles and abstracts following the inclusion and exclusion criteria. Discrepancies were resolved by a third reviewer (radiologist M.E.L. with 10 years of experience). In a second step, the reviewers retrieved the full-text articles of the selected abstracts and performed an independent second-step selection.

### Quality Assessment

The quality of the included studies was evaluated by two readers (radiologists S.C.F. with 3 years of experience and M.F. with 1 year of experience) by using the Quality Assessment of Diagnostic Accuracy Studies-2 criteria, as proposed by Whiting et al (14), and the radiomics quality score (RQS), as proposed by Lambin et al (15).

### Data Collection and Preparation

We collected the relevant data from the included full-text articles into an evidence table (Table). From each publication, we specified the following information: first author’s name, year of the publication, number of patients, tumor grade, type of *IDH* mutation, and whether single or multiple sequences and/or modalities were used for radiomics feature extraction. Two reviewers independently conducted data extraction, and all discrepancies between them were resolved at a consensus meeting.

**Table:**
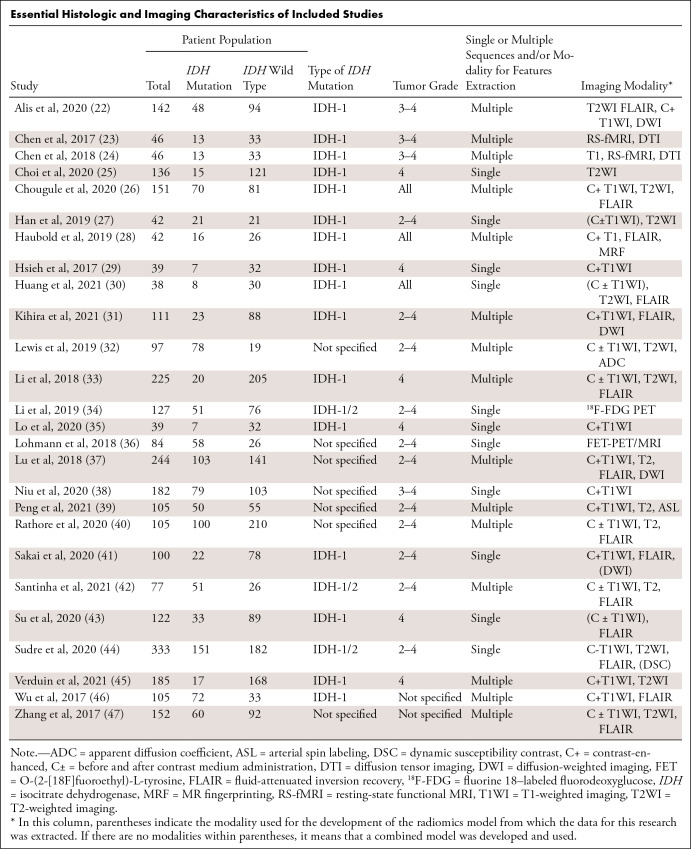
Essential Histologic and Imaging Characteristics of Included Studies

The standard 2 × 2 contingency table was constructed and included the true-positive, false-positive, false-negative, and true-negative values. When the data were insufficient to complete the 2 × 2 table, corresponding authors were contacted through email to request the masked data.

Each study was labeled as “low” or “good” quality based on its overall RQS, using the median value as cutoff. In addition, studies with RQS of less than 6 were categorized as “very low quality” studies, as compared with “adequate quality” studies. This data-driven categorization was determined by a subset of the RQS distribution that exhibited notably low scores (see Results section). We assessed tumor grade homogeneity as an additional factor by categorizing studies into those that incorporated only grade 4 gliomas versus those including all-grade gliomas. Furthermore, we arranged studies according to the acquisition parameters, such as single versus multiple sequences and/or modalities, used for radiomics feature extraction.

Moreover, as we found potential dataset overlap among articles, we conducted a reduced-sample meta-analysis (as a sensitivity analysis [16]) using only the articles with an independent dataset—that is, without any potential overlap among training sets.

### Statistical Analysis

The κ statistics were calculated to assess the agreement between the two raters during the full manuscript review process. We categorized the strength of agreement measured by the κ statistic as no (< 0.0), none to slight (0.0–0.20), fair (0.21–0.40), moderate (0.41–0.60), substantial (0.61–0.80), or almost perfect (0.81–1.0) agreement.

Diagnostic forest plots of sensitivity and specificity were obtained for the included studies. As the forest plot is a univariate analysis, we additionally explored the bivariate relationship and variation between sensitivity and specificity estimates using crosshairs plot (17) and ROCellipse plot (18). Bivariate meta-analysis (19) with a random-effects model was used to estimate pooled sensitivities and specificities across studies. This model jointly analyzes the pairs of logit-transformed sensitivity and specificity from studies, incorporating the inherent correlation between them and reducing the potential bias in the estimate of the CIs and heterogeneity. The summary receiver operating characteristic (ROC) curve was derived, and the area under the ROC curve (AUC) was estimated. The pooled sensitivity and specificity, the positive and negative likelihood ratios, and the diagnostic odds ratios (20) were obtained with the 95% CI estimates.

The χ^2^ tests were performed on the original data to separately assess heterogeneity of sensitivities and specificities. To address the impact of heterogeneity on the meta-analysis (21), we also assessed *I*^2^ and Cochran *Q* of positive and negative likelihood ratios and the associated diagnostic odds ratios (20). Subgroup analyses were performed to qualitatively highlight differences in the pooled outcomes in settings with different RQS values (low or good and very low or adequate, as previously defined above), tumor grades (grade 4 vs mixed grade) and acquisition parameters (single vs multiple sequences and/or modalities used for radiomics feature extraction).

Additionally, meta-regression analyses were performed to quantify the effect of these categorical covariates and of RQS considered as a continuous variable using separate regression models. A meta-regression was also used to investigate the relationship between study sample size and accuracy outcomes. The comparative summary ROC curves were assessed.

*P* < .05 was considered statistically significant. Statistical analyses were performed with the open-source package *mada* (18) written in R software (R Project for Statistical Computing) using RStudio, version 1.4.1106 (Posit) (18).

## Results

### Study Selection

**Figure 1:**
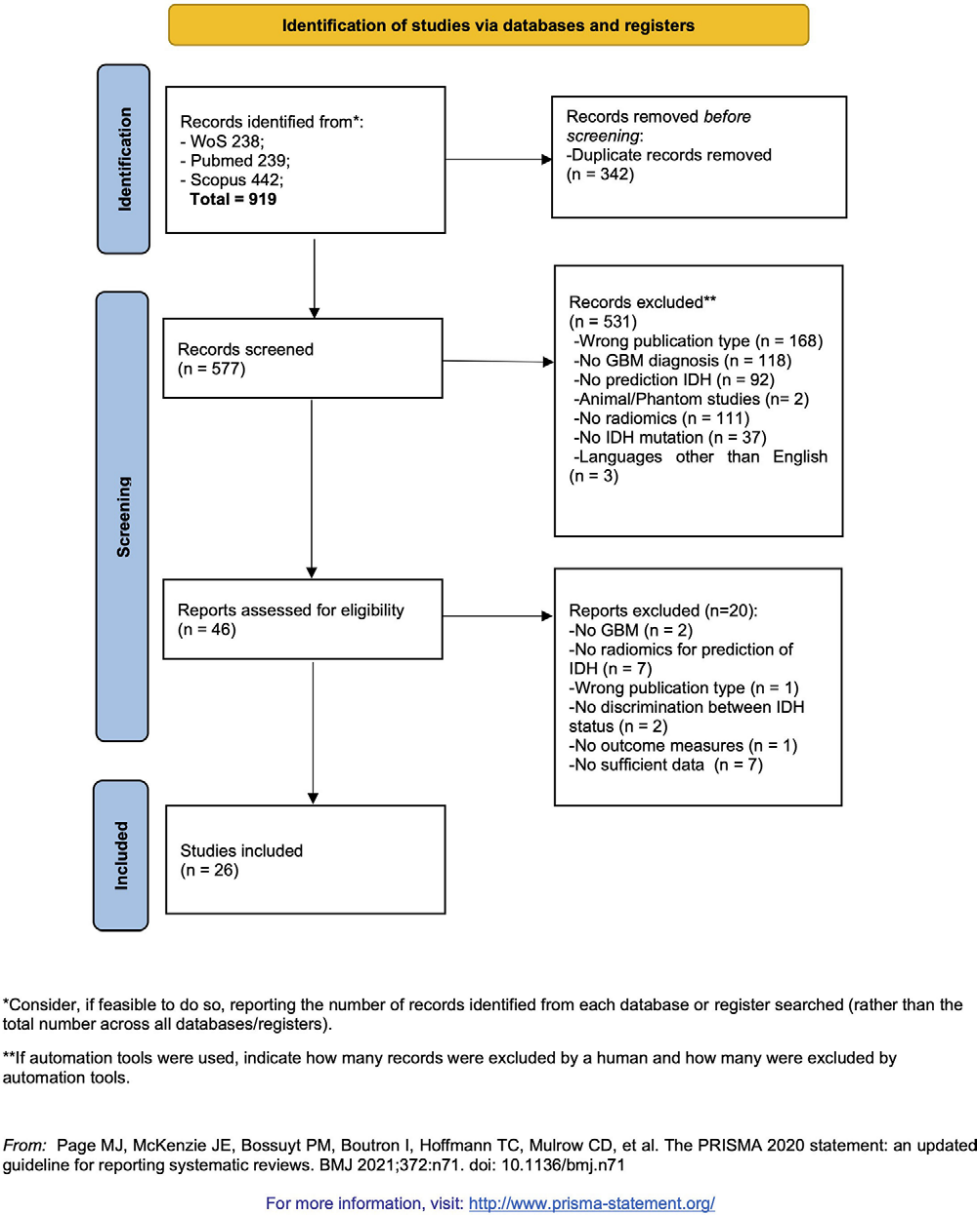
Flow diagram for selection pipeline according to the Preferred Reporting Items for Systematic Reviews and Meta-Analyses 2020 statement. GBM = glioblastoma, *IDH* = isocitrate dehydrogenase, WoS = Web of Science. (Adapted, under a CC BY 4.0 license, from reference 13.)

We initially identified 919 relevant articles. After removing duplicates, 577 titles and abstracts were reviewed, and 531 studies were excluded according to the eligibility criteria. The remaining 46 studies were potentially appropriate and were assessed for eligibility according to the inclusion criteria. After the full-text review, 33 articles were considered eligible for the meta-analysis. During data extraction, we contacted the authors of nine articles to obtain missing data. As we received complete answers in only two cases, we excluded the remaining seven due to insufficient data. Finally, 26 studies were selected for this meta-analysis (Fig 1).

Cohen κ was calculated to assess the agreement between two raters during the full manuscript review process and showed an almost perfect agreement (κ = 0.93).

### Study Characteristics

A total of 3280 patients were described in the 26 included studies, including training, validation, and test sets. The different models were trained on 2527 patients, with a median 82 patients (IQR: 45.5–127). All studies included patients with GBM as defined in the World Health Organization 2016 classification; 18 (69.6%) also included grade 3 gliomas, 14 (53.9%) also included grade 2 gliomas, and three (11.5%) included all four grades of gliomas. In two studies, the grade of lower-grade gliomas was not specified. The isoform of *IDH* tested for mutation was specified in 19 studies (73%): both *IDH1* and *IDH2* in three studies (11.5% [three of 26]) and only *IDH1* in 16 studies (61.5% [16 of 26]).

### Quality Assessment

The total RQS was calculated for each article and each component. The RQS (mean ± SD) was 10.6 ± 3.3 (29.4% of the possible maximum value of 36); the maximum score was 14 (38.9%), and the minimum score was 4 (11%). Importantly, five articles had scores of 4–5, while all the others scored 9 or higher. This observation led to categorization of this subgroup as a very low quality RQS subgroup. The RQS basic adherence rates of the studies were calculated according to the six key domains proposed by Park et al (48). Details about the adherence to the single domains are reported in Appendix S1 (see also Table S1).

Data about the risk of bias and applicability concerns, calculated according to the Quality Assessment of Diagnostic Accuracy Studies-2, for the 26 studies are reported in Appendix S1 (see also Fig S1 and Table S2).

### Diagnostic Accuracy of Radiomics in Predicting *IDH* Mutation

**Figure 2:**
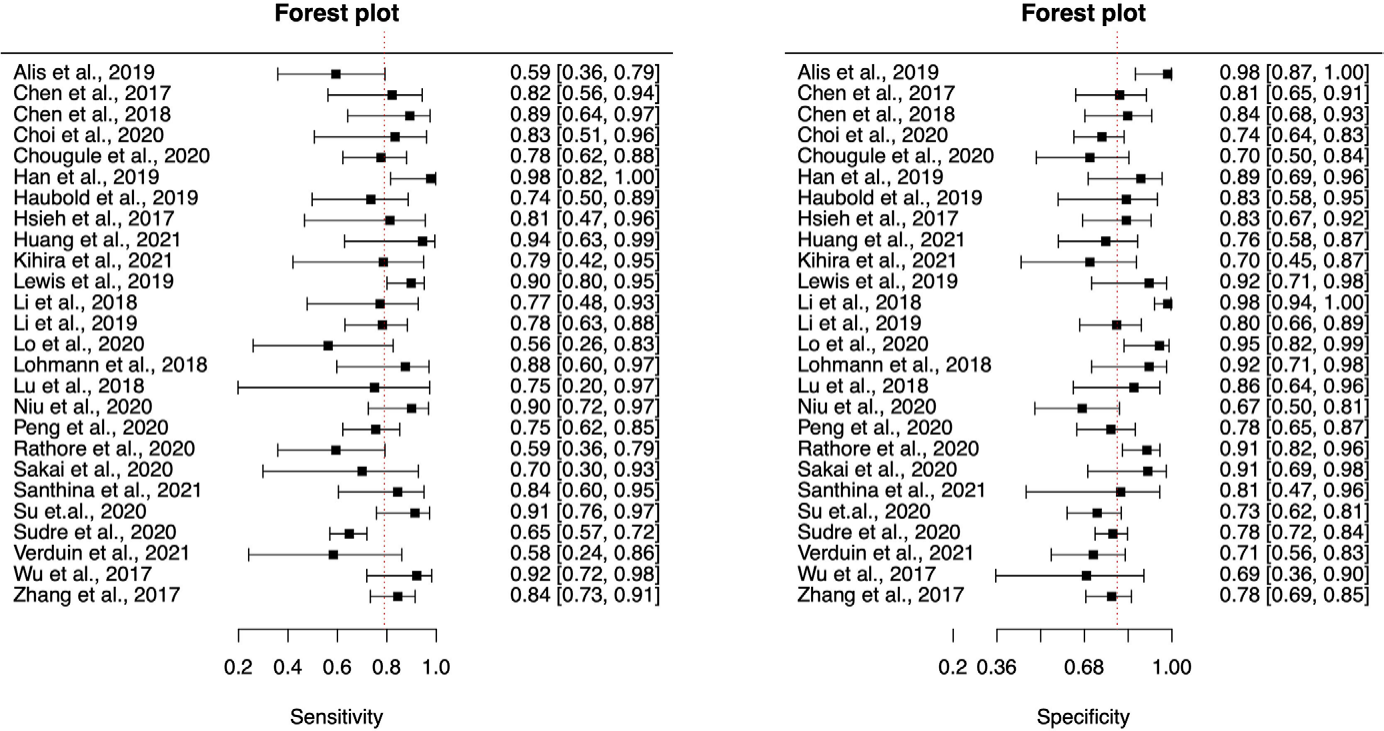
Forest plots of sensitivity and specificity with 95% CIs per study. Vertical red dashed lines denote summary estimates of sensitivity and specificity.

**Figure 3:**
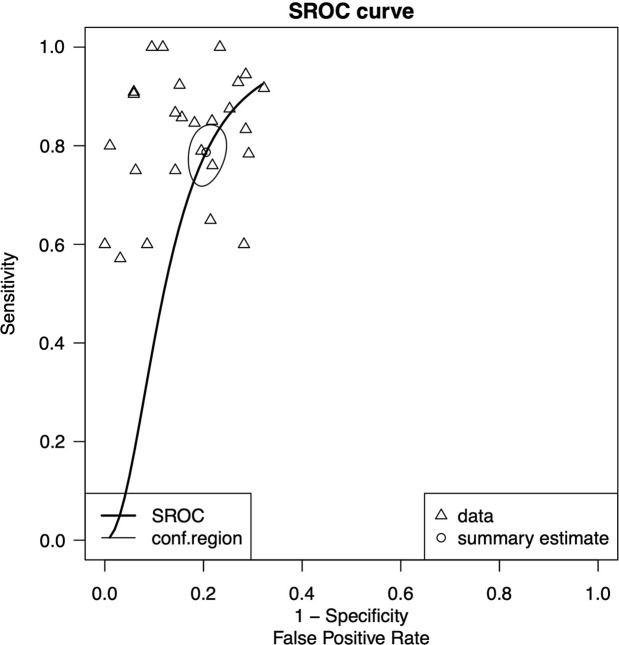
Graph of summary receiver operating characteristic (SROC) curve of pooled sensitivity and specificity of all studies included in the meta-analysis (26 studies), with area under the ROC curve of 0.85, indicating good performance of radiomics analysis for predicting isocitrate dehydrogenase mutation. Conf = confidence.

Paired diagnostic forest plots of sensitivity and specificity, the weighted crosschair plot, and the ROCellipse plot of the 26 included studies are shown in Figure 2, Figure S2A, and Figure S2B, respectively. The pooled sensitivity of radiomics for the detection of *IDH* mutation was 79% (95% CI: 76, 83) with heterogeneity (χ^2^ = 49; *P* < .001), and the pooled specificity was 80% (95% CI: 76, 83) with heterogeneity (χ^2^ = 59.4; *P* < .001). The overall positive likelihood ratio was 3.9 (95% CI: 3.3, 4.6), and the negative likelihood ratio was 0.28 (95% CI: 0.23, 0.34). The pooled diagnostic odds ratio was 17.8 (95% CI: 12, 26). Figure 3 shows the summary ROC curve of pooled sensitivity and specificity with an AUC of 0.85, indicating good performance for the prediction of *IDH* mutation.

### Heterogeneity

The χ^2^ test provided evidence of significant heterogeneity for sensitivity (χ^2^ = 49; *df* = 24; *P* < .001) and specificity (χ^2^ = 59.4; *df* = 24; *P* < .001). For positive and negative likelihood ratio and diagnostic odds ratios, Cochran *Q* was 28.3 (*df* = 25; *P* = .30), 21.7 (*df* = 25; *P* = .65), and 22.5 (*df* = 25; *P* = .61), respectively; Higgins *I*^2^ was 11.5%, 0%, and 0%, respectively.

### Subgroup Analyses

**Figure 4:**
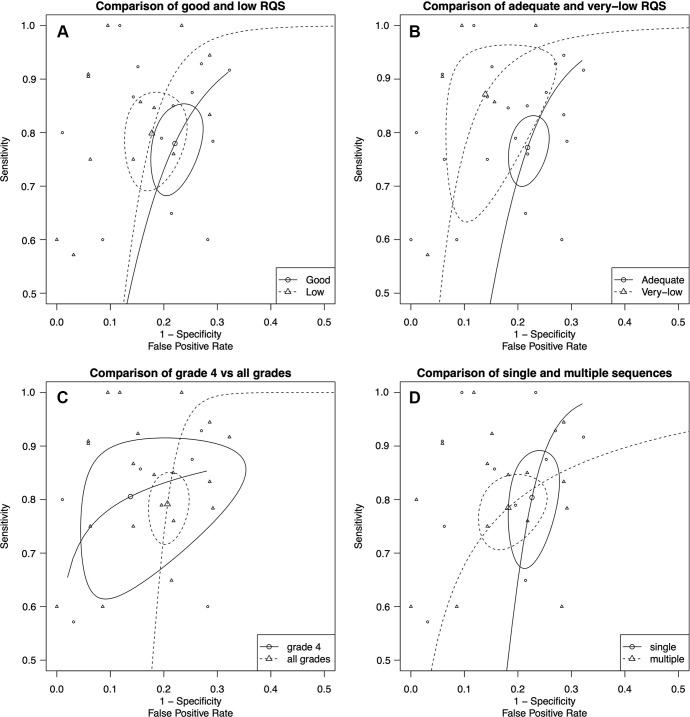
**(A)** Comparison of low and good radiomics quality score (RQS) studies with summary receiver operating characteristic (ROC) curves showing that the summary estimates for both groups are separated, but the confidence regions are overlapped. The area under the ROC curve (AUC) for predicting isocitrate dehydrogenase mutation was 0.85 for low RQS and 0.84 for good RQS. **(B)** Comparison of very low and adequate RQS studies with summary ROC curves showing that the summary estimates for both groups are well separated, but the confidence regions are slightly overlapped. The AUC was 0.92 for low RQS and 0.83 for high RQS. **(C)** Comparison of grade 4 and mixed-grade (2–4) glioma studies with summary ROC curves. Note the overlapping summary estimates and confidence regions for both groups. The AUC was 0.87 for grade 4 gliomas and 0.82 for mixed-grade gliomas. **(D)** Comparison of summary ROC curves for single versus multiple sequences and/or modalities. Note the distinct overlap of the summary estimates and confidence regions. Nonetheless, slightly higher false-positive rates were detected in studies using modalities with multiple sequences. The AUC was 0.88 for single sequence modalities and 0.79 for multiple sequence modalities.

***Radiomics quality score.—*** In low RQS settings (lower half of the distribution, 13 primary studies), the pooled sensitivity for detecting *IDH* mutations was 80% (95% CI: 72, 86) with heterogeneity (χ^2^ = 21; *P* = .048), and the pooled specificity was 83% (95% CI: 78, 87) without heterogeneity (χ^2^ = 17; *P* = .14). In good RQS settings (13 primary studies), the pooled sensitivity for detecting *IDH* mutation was 78% (95% CI: 70, 84) with heterogeneity (χ^2^ = 25; *P* = .02), and the pooled specificity was 78% (95% CI: 74, 82) with heterogeneity (χ^2^ = 39; *P* < .001). Figure 4A depicts the comparison of low versus good RQS, with summary ROC curves showing that the summary estimates for both groups are separated, but the confidence regions overlap. The calculated AUC for low and good RQS was 85% and 84%, respectively.

We conducted additional analyses to compare very low versus adequate RQS studies. In studies with very low RQS (five primary studies), the pooled sensitivity for detecting *IDH* mutation was 87% (95% CI: 69, 95) with heterogeneity (χ^2^ = 11; *P* = .03), and the pooled specificity was 87% (95% CI: 78, 92) without heterogeneity (χ^2^ = 4.4; *P* = .35). In studies with adequate RQS (21 primary studies), the pooled sensitivity for detecting *IDH* mutation was 77% (95% CI: 72, 82) with heterogeneity (χ^2^ = 31.5; *P* = .048), and the pooled specificity was 78% (95% CI: 75, 81) with heterogeneity (χ^2^ = 51; *P* < .001). Figure 4B depicts the comparison of very low versus adequate RQS, with summary ROC curves showing that the summary estimates and confidence regions for both groups are well separated. The AUC was 0.92 for very low RQS and 0.83 for adequate RQS.

Meta-regression using RQS as a continuous covariate showed no evidence of association with the pooled outcomes. Subsequent analyses were carried out based on categorical RQS subgroups. Meta-regression using very low versus adequate RQS as a categorical covariate showed a significant regression coefficient for the false-positive rates (low RQS, *z* = -1.9; *P* = .02), indicating that the false-positive rates are lower for the very low RQS studies and higher for the studies with adequate RQS (Table S3). Meta-regression using low versus good RQS as a covariate showed borderline regression coefficient for the false-positive rates (low RQS, *z* = -1.7; *P* = .085) (Table S3).

***Tumor grade, number of sequences and/or modalities, and patient overlap.—*** The additional subgroup analyses showed no significant association between tumor grade, number of sequences or modalities used, and the pooled sensitivity and specificity. A sensitivity analysis with only the articles without any potential dataset overlap was substantially similar to the comprehensive analysis. Detailed description of these results is available in Appendix S1.

## Discussion

We conducted a bivariate meta-analysis to leverage the pooled sensitivity and specificity of diagnostic accuracy studies that applied radiomics to predict *IDH* mutation status in diffuse gliomas. Our results indicate that radiomics techniques achieve good accuracy performance in distinguishing *IDH* mutation status in patients with diffuse gliomas, with pooled sensitivity and specificity of 81% and 79%, respectively. Tumor grade, number of sequences or modalities, and methodologic quality according to the RQS were identified as potentially prominent sources of variability and were used as criteria for conceptualizing the subgroup analysis. Among these, only the RQS was found to be a potential source of heterogeneity.

Pooled outcomes measured in our meta-analysis were lower than reported in a previous work. In the span of 2 years from the only other meta-analysis about radiomics-based *IDH* prediction in gliomas (12), the number of articles eligible for such analysis has almost tripled, with sensitivity decreasing from 88% to 79% and specificity decreasing from 87% to 80%. A plausible explanation of this decrease might be that our meta-analysis was grade 4 oriented, with systematic exclusion of studies including only grade 1–3 gliomas. Grade 4 gliomas, with a dramatically lower prevalence of *IDH* mutations compared with lower grades (49), might have introduced class imbalance-related issues, with variations in the sampling strategies affecting final accuracy measurements (50). Additionally, the number of articles lacking model validation was four of nine (44%) in Zhao et al (12) and four of 26 (15%) in the present work. A separate validation set is essential to avoid overfitting and outcome metric overestimation. In this respect, the negative association we found between sample size and sensitivity suggests that results are more influenced by overfitting than by the benefit of training set expansion. Last, we performed subgroup analyses using data from training sets, although the sample size was admittedly too low, leading to possibly unreliable conclusions. Contrary to the previous study, our subgroup analysis was conducted using outcomes from validation sets. Comparing the outcomes of pure grade 4 versus all-grade studies suggested that the inclusion of articles with mixed cohorts did not substantially alter the main outcomes. Similarly to Spadarella et al (51), we found globally low RQS scores (15) and hypothesized that methodologic quality might introduce a bias in the pooled outcomes. In the absence of established cutoffs for the RQS, we categorized our cohort based on data-driven hypotheses, and we found that an RQS of less than 6 (corresponding with the very low quality subgroup) predicts higher specificity, suggesting possible overestimation. Despite continuous improvements of the data analysis algorithms, witnessed by the overall increase in adherence to domain 4 of RQS, organizational milestones, such as the availability of multicenter data and prospective study designs, are still insufficient and therefore hamper research quality in radiomics. A last subgroup analysis, based on training data for algorithms, found no macroscopic differences between single and multiple sequence radiomic pipelines, possibly due to the absence of widespread standards for radiomics pipelines and inappropriate data dimensionality reduction techniques (52).

Our results showed that radiomics techniques do not outperform advanced imaging in accurately predicting *IDH* mutational status in gliomas. A meta-analysis by Suh et al (53) showed that a combination of diffusion- and perfusion-weighted MRI allows for the noninvasive prediction of *IDH* mutational status in all-grade gliomas with a summary sensitivity of 86% and specificity of 87%. Sensitivity was further enhanced in a subgroup using 2-hydroxyglutarate MR spectroscopy, which detects a metabolite selectively accumulated in *IDH*-mutant gliomas. Compared with MR spectroscopy, radiomics does not require 2-hydroxyglutarate-specific sequences optimization and complex postprocessing implementation; furthermore, effective algorithms could be widely available in centers without MR spectroscopy expertise. The lack of incremental benefit from sporadic use of perfusion- and diffusion-weighted imaging in the studies included in the present work, compared with the qualitative evaluation of these sequences made in Suh et al (53), should be reevaluated in future studies in light of more extensive evidence. Similarly, good *IDH* discrimination performances have also been reported for nonradiomics artificial intelligence applications, such as deep learning (54) and liquid biopsy (55). Deep learning techniques can achieve high diagnostic accuracy, but current state-of-the-art applications lack explainability and work as “black boxes,” thus raising doubts about biologic significance and generalizability. Unlike radiomics, no distinct features can be isolated, compared across studies, or assessed for potential clinical significance in future research. Likewise, though promising, liquid biopsy must face challenges such as reagents availability in clinical routine and difficulty in obtaining cerebrospinal fluid samples in a preoperative setting (55).

This study had limitations. First, due to the limited number of studies with a pure grade 4 cohort, we included studies with both grade 4 and lower-grade gliomas. Second, substantial variability existed in the imaging protocols and MRI sequences used. Our subgroup analysis oversimplified this variability into a categorical distinction (single vs multiple sequences and/or modality). Additionally, the validation pipeline and machine learning technique to fit the final models were highly variable among the studies. Based on the numerosity of each study sample, different strategies of model validation were used, ranging from leave-one-out cross-validation to independent test sets. Therefore, differential propensity to overfitting is likely to have influenced the estimated effect magnitude. The reliance on the RQS as the only available tool for the quality assessment and the data-driven approach in choosing the cutoffs for the subgroup analysis posed further limitations to our study. Last, there was potential dataset overlap among the included primary studies. Based on the anonymized, geographical information provided by the single studies, we inferred that patients’ data were not used in multiple studies. Yet, eight articles included anonymized data from The Cancer Imaging Archive dataset in their training set. A sensitivity analysis conducted without this subset was not substantially different from the results of our comprehensive database. The literature provides limited evidence regarding a standardized and rigorous method to handle overlapping datasets or patients in meta-analyses, especially in the radiomics field. While recommending caution in the interpretation of the results, we nonetheless chose not to exclude articles with potentially overlapping datasets, both to avoid arbitrary decisions and to preserve the information provided by different radiomic pipelines.

In conclusion, radiomics is a promising tool for the determination of *IDH* mutational status in grade 4 and lower-grade diffuse gliomas. However, in recent years, the performance of radiomics-based algorithms has not improved to the point of overcoming conventional approaches, limiting their widespread use in clinical routine. Missed compliance to several quality criteria is a further remarkable caveat for the translation of the predictive algorithms into clinical practice.
